# Sociodemographic and health factors associated with chronic pain in institutionalized elderly

**DOI:** 10.1590/0104-1169.3552.2510

**Published:** 2014

**Authors:** Maria Helena Barbosa, Alison Fernandes Bolina, Jordânia Lumênia Tavares, Aldenora Laísa Paiva de Carvalho Cordeiro, Raíssa Bianca Luiz, Karoline Faria de Oliveira

**Affiliations:** 1 PhD, Adjunct Professor, Universidade Federal do Triângulo Mineiro, Uberaba, MG, Brazil; 2 Master's students, Universidade Federal do Triângulo Mineiro, Uberaba, MG, Brazil; 3 Doctoral student, Universidade Federal do Triângulo Mineiro, Uberaba, MG, Brazil

**Keywords:** Aged, Chronic Pain, Aging, Nursing, Pain Management

## Abstract

**OBJECTIVES:**

to characterize chronic pain in institutionalized elderly and verify the associated factors.

**METHOD:**

observational, cross-sectional and non-experimental study with a quantitative approach. The study participants were 124 elderly living in Long-Term Care Institutions for the Elderly (LTCIs) in a city in Minas Gerais (Brazil). Approval for the project was obtained from the Research Ethics Committee. The elderly's clinical and sociodemographic variables and pain-related aspects were assessed. The data were analyzed through descriptive statistics and bivariate analysis (chi-squared).

**RESULTS:**

the prevalence of chronic pain corresponded to 58.1%; for more than 10 years (26.4%); in lower limbs (31.9%); characterized as "twinges" (33.3%); 33.3% adopted medication treatment; the pain did not improve (41.7 %); or worsen (34.7 %). It was evidenced that elderly aged 60├ 70 old had 70% less chances of chronic pain than those aged 80 years and older (p=0.018).

**CONCLUSION:**

institutionalized elderly have a high prevalence of chronic pain, mainly in the lower limbs. No factors of pain improvement or worsening were identified and medication was evidenced as the preferred treatment. Age showed to be associated with the presence of pain. It is considered important to accomplish multiprofessional actions at the LTCIs to guide prevention and rehabilitation actions of the pain episodes in these elderly.

## Introduction

Population aging and increased life expectancy have increased the investments and health research focused on this age range, in Brazil and around the world. Estimates from the Informatics Department of the Unified Health System (DATASUS) demonstrate that 11.77% of the population in the State of Minas Gerais are elderly people. According to the same data from DATASUS, in the city of Uberaba (Minas Gerais - Brazil), the place of study, this percentage is higher (12.6%), indicating the need for research in that city to get to know these individuals' health needs^(^ 1 ^)^.

The global population aging phenomenon has led to an increase in the demand for Long-Term Care Institutions for the Elderly (LTCIs). The institutionalization of the elderly generally happens in situations in which these subjects become dependent, entailing the need for help from family and/or third parties, which do not always have structural and socioeconomic resources to attend to them in the best possible manner^(^ 2 ^)^.

Surveys undertaken at LTCIs have observed a high incidence of chronic and degenerative morbidities among elderly people, which are mostly related to chronic pain reports^(^ 3 ^-^ 5 ^)^.

According to the International Association for Pain Studies^(^ 6 ^)^, chronic pain can be defined as an unpleasant sensory and emotional experience caused by an actual or potential tissue injury. Commonly associated with chronic pathological process, it can be continuous or recurring, mild to intense and last more than six months^(^ 7 ^)^.

It is highlighted that chronic pain has multiple causes and can be related to different factors^(^ 8 ^)^. A study of institutionalized elderly in Bahia did not show an association between the presence of chronic pain and sex (p=1.0) and age range (p=0.59)^(^ 5 ^)^. A study of elderly people in São Paulo found that chronic pain was associated with altered physical mobility (p<0.001) and functional disability for basic (p<0.001) and instrumental activities of daily living (p<0.001)^(^ 9 ^)^. In this research, which was developed with a community population, ratified the lack of scientific publications on this theme, especially among elderly people living in LTCIs.

Thus, considering that the prevalence of chronic pain among the institutionalized elderly (45 to 80%) is higher than among community residents (20 to 50%)^(^ 10 ^)^; knowledge on the characteristics and factors associated with chronic pain in this population is necessary for the nurses, in their work at the LTCIs, to be able to propose more specific health care strategies, aiming for its control and treatment.

In view of the above, the following study objectives were outlined: to verify the occurrence of chronic pain in institutionalized elderly, therefore characterizing it according to the time, location, type, most used treatments and factors of worsening and improvement; and to identify the sociodemographic and health factors associated with chronic pain in this population.

## Materials and methods

Observational, cross-sectional field study with a non-experimental design and quantitative approach; undertaken at LTCIs located in the city of Uberaba-MG, Brazil, registered by the Municipal Council of Social Assistance of the Municipal Government. The city of Uberaba has nine registered LTCIs, which are currently sheltering 282 individuals. The study participants were 124 elderly who attended to the following inclusion criteria: accepting to participate in the research and signing the Free and Informed Consent Form, aged 60 years or older and ability to express oneself. In total, 158 (56.3%) residents were excluded from the research because they did not attend to the inclusion criteria: 102 (64.56%) due to limitations to understand the questions and inability to express the answers, 48 (30.38%) had not completed 60 years of age, eight (5.06%) did not accept to participate in the research and eight were not present at the moment of the interview due to an excursion or hospitalization.

To collect the data, initially, appointments were made with the managers of the LTCIs for the researchers to interview and assess the elderly. The data were collected between February and July 2010, using a two-part instrument constructed for this purpose. The first referred to the clinical sociodemographic data; the second to the pain-related aspects (onset, location, factors of improvement and worsening and adopted treatments). It is highlighted that the data collection instrument used was submitted to face validation of the content. No instrument was identified in the literature that addressed all of the aspects investigated in this study. Nevertheless, there are chronic pain assessment instruments in the literature, which did not consider all of the variables of interest in this research though.

To identify the elderly with chronic pain, the participants were considered who referred this symptom with duration of six months or more, according to the definition proposed by the North American Nursing Diagnosis Association (NANDA)^(^ 7 ^)^.

The data were inserted in an electronic worksheet in the software Excel*(r)* for Windows*(r)*, later validated through dual input and exported to the software Statistical Package for the Social Sciences (SPSS), version 19.0, for processing and analysis.

The qualitative variables were analyzed according to descriptive statistics, using absolute and percentage frequency distribution. For the quantitative variables, descriptive measures of centrality (mean) and dispersion (standard deviation, minimum and maximum) were used.

To verify the factors associated with chronic pain, initially, preliminary bivariate analysis was applied, using the chi-square test. The predictive variables were sex, age range, regular physical exercise, presence of self-referred morbidities and impaired physical mobility. Variables with *p*<0.1 were considered as statistically significant associations. Next, multivariate analysis through logistic regression was used, with a statistical significance level of 0.05.

Approval for this study was obtained from the Research Ethics Committee at Universidade Federal do Triângulo Mineiro, under Opinion 1360/2009, in compliance with the ethical principles for research involving human beings, according to Resolution 196/96.

## Results

### Sociodemographic and health characteristics 

A predominance of female elderly was observed, aged 70├ 80 years and single. As regards the education level, the majority indicated primary education, followed by no education. Individuals with children were predominant.

Concerning the health variables, elderly who referred morbidities were predominant, the most mentioned of which was systemic arterial hypertension (25.9%). Most of the individuals in this research did not practice physical exercise regularly.

Table 1 below shows the sociodemographic and health characteristics of the study population.

**Table 1 -:** Frequency distribution of sociodemographic and health variables of institutionalized elderly. Uberaba, MG, Brazil, 2013 (n=124)

Variables		n	%
Sex			
	Male	61	49.2
	Female	63	50.8
Age range (in years)			
	60├ 70	35	28.2
	70├ 80	46	37.1
	80 and older	43	34.7
Marital status			
	Never married or lived with a partner	49	39.5
	Married	21	16.9
	Widowed	40	32.3
	Separated/Divorced	14	11.3
Education			
	None	46	37.1
	Finished primary education	72	58.1
	Finished secondary education	4	3.2
	Finished higher education	2	1.6
Children			
	No	52	41.9
	Yes	72	58.1
Self-Referred Morbidities			
	No	39	31.5
	Yes	85	68.5
Physical exercise			
	No	110	88.7
	Yes	14	11.3
Chronic pain			
	No	52	41.9
	Yes	72	58.1

A prevalence of 58.1% of chronic pain was verified in the study population. The highest percentage of elderly indicated the onset of pain more than 10 years earlier. 

As regards the pain location, the lower limbs were prevalent, followed by the dorsal region. The most mentioned pain type were "twinges", followed by burning pain.

With regard to the pain treatment, the largest proportion received medication treatment. Nevertheless, 33.3% did not adopt treatments.

Most of the interviewed elderly reported no factor that improved the chronic pain. Concerning the factors that worsened the chronic pain, most of the interviewees also informed that there was no specific factor. The chronic pain characteristics are indicated in Table 2.

**Table 2 -:** Distribution of factors related to chronic pain among the elderly living in the LTCIs. Uberaba, MG, Brazil, 2013 (n=72)

Characteristics		N	%
Onset			
	6 months ├ 1 year	17	23.6
	1├┤ 2 years	14	19.4
	2 ┤4 years	9	12.5
	4 ┤ 10 years	13	18.1
	10 years or more	19	26.4
Location			
	Upper limbs	4	5.6
	Lower limbs	23	31.9
	Back region	11	15.3
	Abdomen	6	8.3
	General	5	6.9
	Joints	4	5.6
	Craniofacial	3	4.2
	Lower limbs and joints	2	2.8
	Lower limbs and back	4	5.6
	Others	10	13.9
Type			
	Burning	13	18.1
	Twinges	24	33.3
	Squeezing	11	15.3
	Stinging	9	12.5
	Pounding	4	5.6
	Cannot explain	7	9.7
	Outros	4	5.6
Treatment			
	None	23	31.9
	Medication	24	33.3
	Physiotherapy	8	11.1
	Both	17	23.6
Factors of Improving			
	None	30	41.7
	Lying down	16	22.2
	Sitting	2	2.8
	Resting	8	11.1
	Moving the body	6	8.3
	Others	10	13.9
Factors of Worsening			
	None	25	34.7
	Sitting	7	9.7
	Walking	16	22.2
	Moving the body	7	9.7
	Emotional (Irritability)	4	5.6
	Others	13	18.1

## Factors associated with the pain

To verify the factors associated with the chronic pain, the variables sex and age range were inserted in the multivariate analysis, which showed statistical significance (*p*<0.05) in the bivariate analysis. Thus, it was verified that the elderly between 60├ 70 years had 70% less change of chronic pain than elderly aged 80 years and older. 

In Table 3, the factors associated with chronic pain in institutionalized elderly are listed.

**Table 3 -:** Preliminary bivariate analysis and multivariable logistic regression model of factors associated with chronic pain. Uberaba, MG, Brazil, (n=124)

Variables		Chronic pain					χ²	p	Regression Analysis	
		No			Yes					
		N	%		n	%			OR* (CI)	P
Sex							5.46	0.019		
	Male	32	61.5		29	40.3			1	-
	Female	20	38.5		43	59.7			0.50 (0.24-1.08)	0.082
Age range							8.17	0.017		
	60├ 70	21	40.4		14	19.4			0.30 (0.12-0.82)	0.018
	70├ 80	19	36.5		27	37.5			0.60 (0.24-1.48)	0.27
	80 and older	12	23.1		31	43.1			1	-
Regular physical exercise							0.25	0.61		
	No	47	90.4		63	87.5				
	Yes	5	9.6		9	12.5				
Self-referred morbidities							1.07	0.3		
	No	19	36.5		20	27.8				
	Yes	33	63.5		52	72.2				
Impaired physical mobility							1.16	0.28		
	No	26	50.0		29	40.3				
	Yes	26	50.0		43	59.7				

* Adjusted odds ratio

## Discussion

### Sociodemographic and health characteristics 

In this research, the predominance of elderly between 70 and 80 years of age does not follow the pattern found in another study undertaken in LTCIs in Minas Gerais, in which the highest percentage is aged 80 years or older^(^ 11 ^)^.

In line with other studies in Minas Gerais, a predominance of female elderly has been found in the institutionalized population (59.0%)^(^ 11 ^)^ (64.75%)^(^ 12 ^)^. A different result was observed in a study conducted at LTCIs in a city in the State of São Paulo though, where most of the investigated elderly were male (59.13%)^(^ 2 ^)^. This divergence can be related to the social and cultural characteristics of the study groups, as the studies were developed in distinct places in the country.

As regards education, most of the institutionalized elderly only finished primary education, followed by elderly without education. This fact may reflect the difficult access to schools and the educational policies in the past^(^ 13 ^)^.

The result of this research with regard to the marital status was equivalent to a study about the profile of elderly residents at LTCIs, with a predominance of single (63.0%), followed by widowed (23.0%) elderly. It is highlighted that most of the elderly indicated having children, but the institutionalization among these individuals may be related to the financial limitations of their relatives and the absence of a woman from the home, deriving from their greater insertion in the job market, thus making the care for elderly people at home more difficult^(^ 11 ^)^.

As regards the health variables, the institutionalization can contribute to the emergence of diseases^(^ 14 ^)^. It should be highlighted that systemic arterial hypertension (SAH) as the most mentioned disease in this study, reflects a global trend in the elderly population^(^ 15 ^)^. In line with this finding, data from the demographic census in Brazil (53.3%)^(^ 16 ^)^ and a study developed at LTCIs in the city of Marília/SP (64,34%)^(14) ^also observed SAH as the most frequent morbidity in the elderly.

At the LTCIs studied, only a small part of the elderly indicated regular physical exercise. This characteristic may derive from the individuals' limitations or indicate infrastructural and human resource shortages to plan activities appropriate to the age range in the environment they live in.

As regards the occurrence of chronic pain reports, studies undertaken at LTCIs, involving elderly living in Jequié-BA (73.3%)^(^ 5 ^)^ and Spain (61.0%)^(^ 8 ^)^ found higher percentages. Nevertheless, a studied conducted among elderly at an lTCI in Porto Alegre found pain in only 12.7%^(^ 17 ^)^. The lower percentage in that study^(17) ^may be related to the method, which used records in patient histories of the Nursing Diagnoses identified and registered by nurses in the institution. Due to subjective reasons related to the assessment or dependence on disciplined records to reflect the reality, the chronic pain diagnosis may have been undernotified.

Concerning the onset of the pain, the results found diverge from a study undertaken in Jequié-BA, in which the onset between one and five years earlier was the most frequent^(^ 3 ^,^ 5 ^)^. It is highlighted that part of the elderly indicated pain for more than ten years, which is noteworthy, as untreated pain can result in depression, functional disability, sleep disorders, late rehabilitation, malnutrition and cognitive dysfunction^(^ 17 ^)^. In that perspective, different actions can be undertaken to prevent the development of pain, such as health education for self-care, physical exercise and healthy eating^(^ 18 ^)^.

Concerning the pain location, data in this research support the study developed with elderly at LTCIs in Jequié-BA, in which chronic pain in the lower limbs was predominant (47.7%), followed by lumbar pain (25%), but with a lower prevalence^(^ 5 ^)^.

A study in Londrina (PR) showed different findings in the community and at LTCIs, showing the prevalence of pain in the lumbar region (21.7%) and lower limbs (21.5%)^(^ 9 ^)^.

The different in pain prevalence sites may be related to characteristics of the activities of daily living (ADLs) among institutionalized elderly or those living in the community. A study of elderly at LTCIs indicates that institutionalization is often related to physical dependence. In addition, the institutionalization discourages the elderly's independence of the accomplishment of ADLs, their earlier habits are replaced by the institutional caregivers doing these activities for them^(^ 19 ^)^. The discouragement of activity reduces the elderly's functioning, which can give rise to pain.

The predominance of lower limb pain can be related to the cartilage degeneration deriving from biological aging or the occurrence of chronic-degenerative diseases like arthritis and arthrosis^(^ 3 ^)^. Similarly, the aging process can cause bone alterations, besides modifications in the disc and joint parts of the spinal cord; predisposing to back pain in the elderly^(^ 20 ^)^. In that sense, physical and motor stimulation activities need to be encouraged, such as the accomplishment of daily self-care tasks, environmental care or small physical exercises, such as walks on the LTCI patio, activities with impact-reducing and stretching devices. The need is highlighted here for nursing to work with a multiprofessional team, aiming for the accomplishment of such activities in accordance with each elderly's adaptive ability.

The most mentioned pain type was "twinges", differently from data found in the literature, which reveal reports of 40.7% of painful feelings. The same study, however, shows 37% of "burning" pain reports, which is the second most mentioned type in this research^(^ 21 ^)^.

Concerning the treatment type, the results found in this research support the findings from a study undertaken in Spain, in which 44.5% of the elderly mentioned medication use^(^ 8 ^)^. Nevertheless, in a study undertaken in Bahia, it was observed that only 15.9% used medication for pain control^(^ 5 ^)^.

The goals of pain treatment are to specifically treat its causes, reduce the intensity, improve the patients' functional ability, sleep, mood and socialization. It can be through medication, psychological, psychiatric, rehabilitation or functional neurosurgical. Pharmacological and non-pharmacological strategies should be associated to control the pain with lower drug doses, which will reduce the side effects^(^ 22 ^)^. In the context of the LTCIs, the nurses, who have a global view of the elderly and spend more time with them, should articulate the actions with other health professionals, stimulating interdisciplinary actions. Treatments with the same target achieve faster results than professionals' isolated activities.

Concerning the reported factors of improvement and worsening, a study undertaken in Londrina (PR) that involved chronic pain patients attended in a Primary Health Care service found results that differ from this research, with physical exercise as the most mentioned triggering factor (38.51%); and medication use as a pain relief factor in 80.4% of the cases^(^ 9 ^)^. A study undertaken at a multiprofessional pain clinic of a public teaching hospital in Minas Gerais suggests that the perceived pain and intensity are subjective data and that, therefore, the factors that improve and worsen it are directly related with this condition and with the specific pathology involved^(^ 23 ^)^. Nursing should consider the importance of assessing all pain characteristics and their aspects to develop strategies that minimize triggering factors and promote pain improvement factors, thus reducing its manifestations in the elderly's life.

### Factors associated with the pain 

Concerning the factors associated with the chronic pain, the research data support the data found in a study undertaken in Salvador-BA^(^ 24 ^)^, which revealed a statistical association with age. Nevertheless, different studies found other associated factors, like the study undertaken in Bahia with institutionalized elderly, which revealed that sex, age range and length of institutionalization do not influence the presence of pain in the elderly^(^ 3 ^)^.

A study undertaken in Spain in 2007, in turn, indicated that the presence of pain is correlated with the functional ability^(^ 8 ^)^. The fact that younger elderly have a lesser chance of feeling pain than elder elderly may be related to the aging process itself, which is commonly associated with the appearance of chronic illnesses and disability^(^ 18 ^)^. In that sense, the nurses' actions at the LTCI should be linked with the control and monitoring of chronic diseases, aiming to avoid their effects on aging, and with constant pain assessment in order to minimize its impact over the years.

Pain figures among the main factors that limit the elderly's possibility of maintaining a normal daily life, with a negative impact on their quality of life, on the accomplishment of ADLs and, in some situations, restricting social life, leading to isolation^(^ 22 ^,^ 25 ^)^.

## Conclusion

In conclusion, single and female institutionalized elderly between 70├ 80 years of age were predominant. The majority had finished primary education and indicated having children. The morbidity the elderly mentioned most was SAH and they were characterized by a lack of physical exercise.

More than half of the elderly indicated chronic pain, having started more than ten years earlier, mainly in the lower limbs, followed by back pain, twinges and burns, without identifying factors of improvement or worsening, and medication as the preferred treatment. It is highlighted that most of the elderly were not treated for chronic pain. The age range was associated with the presence of pain, with younger elderly between 60├ 70 years old having 70% less chance of chronic pain when compared to those aged 80 years and older.

The survey indicates the importance of further attention to institutionalized elderly's painful symptoms, especially among elder elderly, as these individuals commonly live with pain and do not receive appropriate treatment. And this is mainly due to the health professionals' lack of valuation of these symptoms, as they consider them as characteristic of the aging process.

It is fundamental for nurses, in their work at LTCIs, to have a critical view of the pain complaints, aiming for the appropriate assessment of chronic pain. Pain assessment can contribute to the orientation of care, which can grant relief and comfort for this problem to the elderly. Nevertheless, chronic pain is a complex and multidimensional phenomenon that requires interdisciplinary actions for its control. In the context of the LTCIs, nurses, as active team members, should be able to stimulate these actions, thus contributing to the effectiveness of chronic pain management strategies.

The following limitations should be highlighted: 1) the cross-sectional design, which does not permit the establishment of a causal relation, due to difficulties to identify the process in the analysis of the research variables over time; 2) the restrictions of the research groups concerning the population losses.

Despite these losses, the results can be generalized to the institutionalized elderly, as all elderly living at LTCIs in the city who attended to the inclusion criteria were interviewed. Concerning the research design, it is highlighted that epidemiological and cross-sectional studies are fundamental to support health professionals' more assertive actions.

This kind of studies could be developed on a wider scale, preferably multicenter projects, considering the growth of the elderly population in Brazil and around the world.
